# Willingness to pay for hepatitis B virus vaccine and associated factors among households in Bahir Dar City, northwest Ethiopia: using contingent valuation method

**DOI:** 10.3389/fpubh.2023.1058026

**Published:** 2023-07-05

**Authors:** Addis Aychew, Amare Minyihun, Chalie Tadie Tsehay, Tsegaw Amare, Andualem Yalew Aschalew

**Affiliations:** ^1^Addis Alem Primary Hospital, Bahir Dar, Ethiopia; ^2^Department of Health Systems and Policy, Institute of Public Health, College of Medicine and Health Sciences, University of Gondar, Gondar, Ethiopia

**Keywords:** contingent valuation method (CVM), willingness to pay (WTP), hepatitis B vaccine (HBV), Tobit, Ethiopia

## Abstract

**Background:**

The prevention of disease burden and death through vaccination is one of the most cost-effective methods. Even though the Hepatitis B Virus (HBV) has significant public health problems in Ethiopia, there is no compulsory HBV vaccination program for adults and the vaccine's market value was not yet estimated in the Ethiopia context. Therefore, this study aimed to assess the willingness to pay (WTP) for the HBV vaccine and its associated factors among households in Bahir Dar City, northwest Ethiopia.

**Methods:**

A cross-sectional study was conducted among 620 households from March 1 to 30, 2020. A systematic random sampling technique was employed to select the required number of households. An interviewer-administered questionnaire was used to collect the necessary information. The contingent valuation method was conducted to measure WTP for the HBV vaccine. A Tobit regression model was employed to investigate significantly associated factors, and variables with a *p*-value of <0.05 were considered statistically significant.

**Results:**

In this study, 62.17% of households were willing to pay for the HBV vaccine with an average cost of ETB174.24 (US$5.25). Male household heads (*P* = 0.014), favorable attitude (*P* = 0.017), and good knowledge (*P* < 0.001) toward the vaccine were positively associated with WTP, whereas age (*P* < 0.001), single marital status (*P* = 0.012) and divorced/widowed (*P* = 0.018) marital status were negatively associated with WTP.

**Conclusions:**

Overall, most households were willing to pay for the HBV vaccine with an average demand of ETB174.24 (US$5.25). Therefore, a national-level HBV vaccine strategy should be designed considering the households' willingness to pay. In addition, working on attitudes and knowledge toward the vaccine could potentially increase the household's willingness to pay for the HBV vaccine.

## Introduction

Even though hepatitis B virus (HBV) infection is 50–100 times more contagious than human immunodeficiency virus (HIV) (1), less attention was paid worldwide. Hepatitis B infection is caused by HBV that attacks liver resulting in hepatocellular necrosis and inflammation (2). Worldwide, more than two billion people were infected by the virus, and there were about 620,000 HBV-related deaths each year, with one in four progressing to liver disease (3, 4). Globally, the virus causes 60–80% of all primary hepatocellular carcinoma, which is one of the top three causes of cancer death in sub-Saharan Africa (5). In Ethiopia, 12% of medical admissions and 31% of medical wards' mortality is attributable to liver disease (6).

The prevention of disease burden and death through vaccines is one of the most cost-effective methods (7, 8). Hepatitis B vaccine is designed by the World Health Organization (WHO) as the foremost strategy for preventing death and chronic infection that leads to liver cirrhosis or hepatocellular carcinoma (9). The government of Ethiopia added HBV and Hemophilus influenza type B vaccines to the standard expanded program on immunization (EPI) in 2007 targeting children under 1 year of age (10). On the other hand, the adult vaccination of HBV is only given to high-risk groups, such as healthcare workers in public health facilities, and there is no compulsory HBV vaccination program for adults in the rest of the population and the affordable market cost of the vaccine was not set in Ethiopian context. However, setting the evidence on the willingness of households to pay for the vaccine and their cost ability to pay is very crucial for designing a sustainable program as the cost of the vaccines in Ethiopia is largely covered by unpredictable and unharmonized donors' funds (10).

Studies conducted in Malaysia (11), China (12), and Vietnam (13) showed that 37.5% of the households, 35.6% of migrant workers, and 80.8% of reproductive-age women were willing to pay for HBV, respectively. Moreover, participants in Malaysia (11) and China (12) were willing to pay an average cost of US$73 and US$ 4.65, respectively. Another study conducted in Gondar City, Ethiopia, showed that 62.4% of health professionals were willing to pay an average price of US$ 14.39 for the HBV vaccine (14).

Evidence also showed that various factors contribute to an individual's willingness to pay (WTP) and the amount of money for HBV. Sex, age, marital status, occupation, history of exposure to risky behavior, monthly income, awareness about HBV infection, level of education, perceived susceptibility, having children, adequate information concerning the vaccine, attitude, and knowledge level were among the determinants of people's willingness to pay for HBV (12, 15–24).

Taking its effect on the prevention of HBV infection, growing interest has been noted in determining its value from the consumers' perspective. As there is no current adult HBV vaccine program, estimation of demand for vaccination and associated factors are expected to be useful for the later initiation of the program. Moreover, the finding of this study will help to generate evidence to estimate the national-level market value of HBV vaccination. Besides, it provides insights to help the stakeholders to make decisions that serve the immense interest of the community. Finally, it will serve as a baseline for researchers for further investigation. Therefore, this study aimed to assess the willingness to pay for the hepatitis B virus vaccine and its associated factors among households in Bahir Dar City, Northwest Ethiopia.

## Methods

### Study design and setting

A community-based cross-sectional study was conducted on urban dwellers of Bahir Dar City from March 1 to 30, 2020. Bahir Dar is the capital city of Amhara National Regional State, located 565 KM northwest of Addis Ababa, the capital of Ethiopia. According to the Central Statistical Agency (CSA), the projected population size of the city is estimated to be more than 214,691 in 2017 (25). About 54% of males and 25% of females in Bahir Dar city can fully read and understand. Besides the average monthly income of the households in Bahir Dar city is 4,500 ETB (US$136). The city has six sub-cities and four satellite towns and has a total number of 80,252 households (HHs) (26). It has three public and four private hospitals. Moreover, there are ten health centers and more than 30 private clinics.

### Population, sample size determination and sampling technique

The source population of this study was all urban household heads who live in Bahir Dar City whereas the study population was all urban household heads who live in the selected sub-cities and satellite towns. A total of 620 household heads who lived in the selected sub-cities and satellite towns for at least 6 months were included in the study.

Using the formula *n* = (Zα/2)^2^σ^2^/d^2^ and assuming US$73 as the mean amount cost that households willing to pay for HBV vaccine (11), 95% confidence level, 4% of the mean as a margin of error and σ = 25. After adding a 10% non-response rate and a design effect of 2, the final sample size became 620.

**Figure 1:**
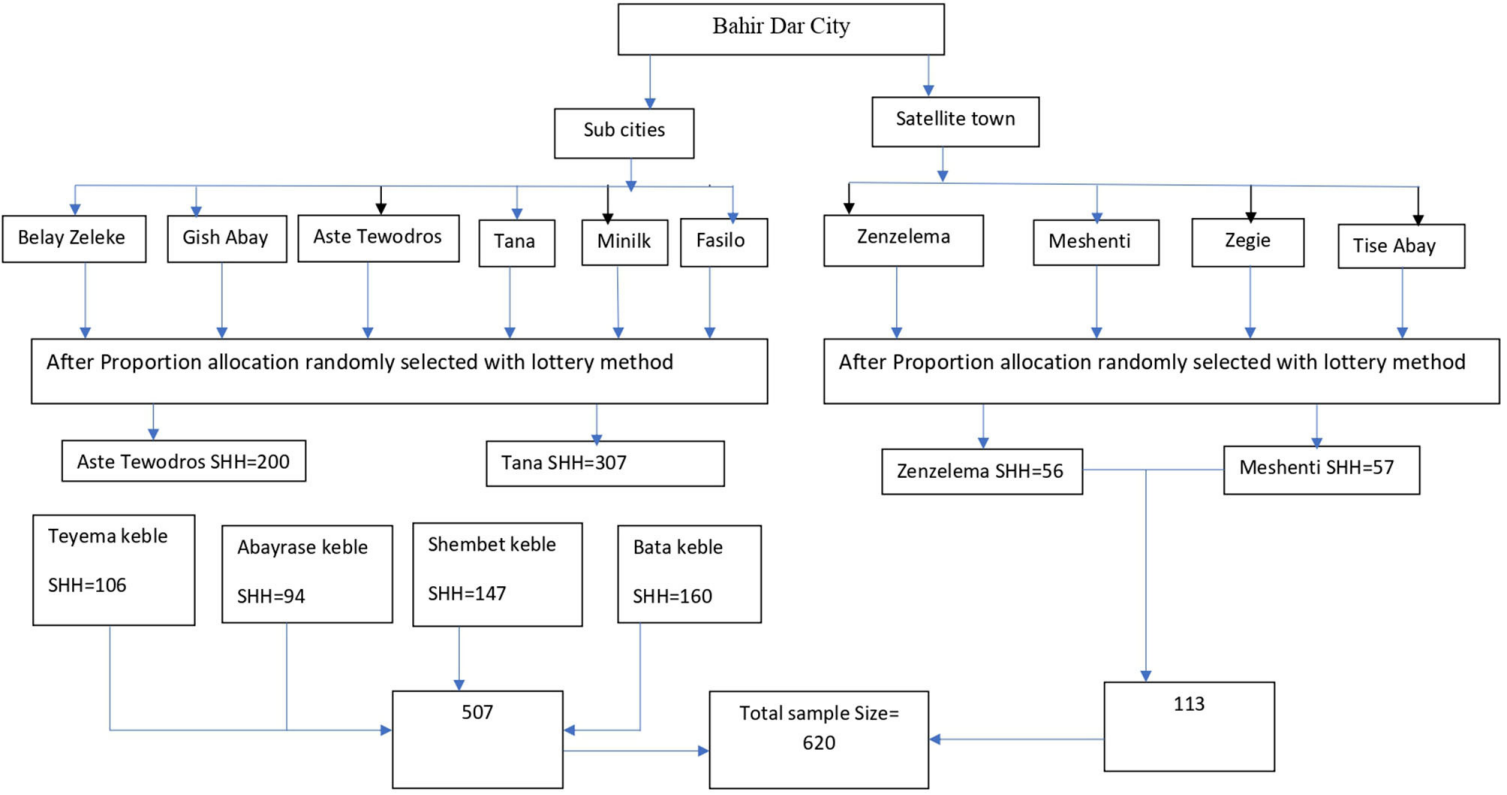
Flowchart for multistage sampling procedures of households in Bahir Dar City urban dwellers, northwest Ethiopia, 2020.

Multistage sampling followed by a systematic random sampling technique was used to select the required number of HHs after the proportional allocation was applied to select HHs in each kebele. Two sub-cities and one satellite town were chosen randomly among six sub-cities and four satellite towns, respectively. Then four kebeles from the two sub-cities and one satellite town were selected randomly using the lottery method (Figure 1). The final number of HHs was selected by systematic random sampling with a k value of 15. When the selected household is closed, again it will be checked for the second time, and if it is closed again the next household will be selected and the sampling pattern will keep on the second household.

### Study variables

The dependent variable of this study was the household's willingness to pay for HBV, which is the household heads' willingness to pay for the HBV vaccine. It is measured in terms of proportion for their acceptance to take the vaccine (yes/no) and if yes, the average number that the HH head is willing to pay for the vaccine will be determined. Whereas, the independent variables were sociodemographic factors (age, sex, marital status, religion, ethnicity, education status, household family size, wealth status, and occupation), knowledge-related factors (knowledge toward HBV, attitude toward HBV, information about HBV infection and vaccine, source of information toward HBV), and HBV risk-related factors (family history of hepatitis B infection, multiple sexual partners, blood transfusion, and tattooing). Knowledge toward the HBV vaccine was measured with nine yes/no questions and those who answered 50% and above were judged as having good knowledge and the remained as poor knowledge. Attitude toward the HBV vaccine was measured by four questions with five Likert scales and those who answered 50% plus were judged as having a favorable attitude.

### Eliciting the households toward a willingness to pay for the HBV vaccine

This study employed the contingent valuation method (CVM) to elicit the preference of households toward HBV using a double-bound dichotomous choice and bidding game approach. The CVM refers to a non-market valuation method used to estimate the value of goods placed by individuals by using stated preference information (27). In this study, double-bounded dichotomous choice: a simple yes/no question was used to assess the household's willingness to accept the HBV vaccine. The respondents were told about a hypothetical HBV vaccine with scientifically proven attributes and features in such a way that the respondents suppose that the HBV has 95% effectiveness and no side effects. If respondents accept to take the supposed HBV vaccine, they were asked if they were willing to pay 300 ETB to take the vaccine. If the respondent replied “*yes*” to this question, then they were asked if they were willing to pay a 100 ETB added value. After that, similar questions with the price of 100 ETB addition were asked until they refuse to pay. If the respondent answered “*no*” to the initial question (300 ETB), they were asked to pay the same question with a price of 200 ETB, then 100 ETB. A participant who refused to pay at the lowest bid (i.e., 100 ETB) was considered not willing to pay. Finally, the respondents had to answer an open-ended question about the maximum price they would be willing to pay (28).

### Data collection tools and procedure

An interviewer-administered questionnaire was used to collect the data. The tool was developed after the literature review of the related studies (12, 14, 29). It was first prepared in English, then translated to Amharic (the local language), and was translated back to English to check for its consistency with a language expert. A pre-test was conducted among 31 (5% of households) in Gondar town, a city 173 KM far from Bahirdar, and necessary adjustments were made based on the pre-test results regarding questions clarification, adjustment and coherence. A day of training was given for seven data collectors who have a nursing diplomas and two supervisors with a public health degree. Each day after data collection, the principal investigator checked the collected data for its completeness and consistency.

### Data processing and analysis

Cleaned data were entered into Epi Info version 7 and exported to Stata version 14 for further analysis. Descriptive statistics such as mean, standard deviation, frequency, and percentage were presented using tables. Tobit model analysis was used to identify factors that had an association with the mean price of HBV vaccine that households were willing to pay. It has been argued that when the nature of the data is continuous with censoring at zero, the most appropriate estimation technique is the limited dependent variable with the Tobit model (30). First, a bi-variable analysis was done to select factors for the final model. Variables at a *p*-value <0.2 were selected for the final Tobit model checking the assumptions of normality and homoscedasticity of error terms. Then *p*-value with a 95% confidence interval (CI) was reported. Independent factors with a *p*-value <0.05 were considered statistically significant. Finally, the marginal effect that the expected WTP value conditional on being uncensored, E(WTP|WTP>0), was estimated (31). The exchange rate was US$1 = ETB 33.18 (32).

### Ethical considerations

This study was conducted following the Declaration of Helsinki. An ethical clearance letter (Ref No/IPH/837/6/2020) was obtained from the Ethical Review Committee of the Institute of Public Health, University of Gondar. A permission letter from the Bahir Dar City Health Office. The purpose and importance of the study were explained in the consent form, including the right to withdraw from the study if they face any inconvenience. After assuring the confidentiality nature of responses, written informed consent was obtained from each participant.

## Results

### Socio-demographic characteristics of the respondents

A total of 608 participants were interviewed making the response rate 98.06%. The mean (±SD) age of the respondents was 37.67 (±12.88) years. The majority (66.12%) of the participants were female, and more than half (55.92%) were married. Out of the participants, (14.14%) had a family history of hepatitis B infection and the majority (43.26) of the participants had a medium wealth status (Table 1).

**Table 1:** Socio-demographic characteristics of the households in Bahir Dar City, northwest Ethiopia 2020 (*N* = 608).

**Variables**	**Frequency**	**Percent**
Age in a year: mean (SD)	37.67	12.88
**Sex**		
Male	206	33.88
Female	402	66.12
**Ethnicity**		
Amhara	590	97.04
Oromo	10	1.64
Tigray	8	1.32
**Marital status**		
Married	340	55.92
Divorced/Widowed	145	23.85
Single	123	20.23
**Religion**		
Orthodox	535	87.99
Muslim	51	8.39
Protestant	22	3.62
**Educational status**		
Unable to read and write	51	8.38
Primary	158	25.99
Secondary	169	27.80
Diploma	109	17.93
Degree above	121	19.90
**Occupation**		
Civil servant	162	26.64
Self-employers	155	25.49
Merchant	76	12.50
Unemployed	47	7.73
Housewife	149	24.51
Other	19	3.13
**Wealth status**		
Poor	152	25.00
Medium	263	43.26
Rich	193	31.74
**Knowledge: mean (SD)**	3.77	1.85
Poor	12	2.9
Good	401	97.1
**Attitude: mean (SD)**	15.61 (2.37)	
Unfavorable	60	9.9
Favorable	548	90.1
**Household family size: mean (SD)**	3.26 (1.49)	
**Family history of hepatitis B infection**		
Yes	86	14.14
No	522	85.86
**Information about HBV infection and vaccine**		
Yes	467	76.81
No	141	23.19
**Source of information (*****n*** = **467)**		
Television/radio	186	39.83
Health professional	104	22.27
Social media	90	19.27
Friend	78	16.70
Others	9	1.93
**Multiple sexual partners**		
Yes	85	13.98
No	523	86.02
**Tattooing on body**		
Yes	107	17.60
No	501	82.40
**Blood transfused**		
Yes	62	10.20
No	546	89.80

HBV, hepatitis B virus; SD, standard deviation.

### WTP for HBV vaccine

The majority (62.17%) of HHs were willing to pay for the HBV vaccine. The mean (SD) amount of money HHs willing to pay was ETB 174.24 (US$5.25) ± 145.44 (US$4.38); of participants who were willing to pay (9%) were agreed to pay more than ETB 300($9.04) (Table 2).

**Table 2:** Amount of money HHs WTP for HBV vaccine in Bahir Dar City, northwest Ethiopia 2020 (*N* = 608).

**Variable**	**Amount of money in ETB**	**Frequency (%)**
WTP for HBV vaccine *N* = 378	10–150	219 (57.94)
	151–300	125 (33.06)
	301–500	21 (5.56)
	500 above	13 (3.44)

ETB, Ethiopian birr; HBV, hepatitis B virus; WTP, willingness to pay.

### Factors associated with WTP for HBV vaccination

In multiple variable analyses sex, age, marital status, attitude, and knowledge were significantly associated with WTP for the HBV vaccine at a *p*-value of <0.05. Model assumptions of normality and homoscedasticity of error terms were checked and fulfilled. Male respondents were willing to pay higher than females (*P* = 0.014). The amount of money that participants willing to pay were decreased with age (*P* < 0.001); marital status: single participants (*P* = 0.012) and divorced/widowed (*P* = 0.018) were willing to pay a lower amount of money than married individuals, but a good attitude (*P* = 0.017) and knowledge (*P* < 0.001) toward HBV infection and vaccine were associated with higher amount of contribution (Table 3).

**Table 3:** Tobit regression analysis on factors influencing WTP for HBV vaccine in Bahir Dar City, northwest Ethiopia, 2020.

**Variables**	**Coef**	**SE**	***P*-value**	**95% CI**
**Sex**				
Male	61.09	24.77	0.014	12.43, 109.74
Female				
**Age**	−6.14	1.09	0.000	−8.28, −3.99
**Family history of HBV infection**				
Yes	45.97	31.28	0.142	−15.45, 107.40
No				
**Information about HBV**				
Yes	−37.93	32.25	0.240	−101.27, 25.40
No				
**Tattoo on the body**				
Yes	−45.33	30.42	0.137	−105.06, 14.41
No				
**Attitude**	12.35	5.14	0.017	2.25, 22.44
**Knowledge**	49.55	8.04	0.000	33.76, 65.35
**Marital status**				
Single	−74.54	29.46	0.012	−132.40, −16.68
Divorced/widowed	−70.52	29.72	0.018	−128.88, −12.16
Married				
**Education status**				
Primary	−29.63	45.94	0.519	−119.86, 60.59
Secondary	−52.87	46.30	0.254	−143.81, 38.07
Diploma	−78.88	50.35	0.118	−177.76, 20.00
Degree and above	13.27	51.18	0.795	−87.24, 113.79
Unable to read and write				
**Wealth status**				
Poor	32.61	29.70	0.273	−25.72, 90.94
Medium	−47.71	26.60	0.073	−99.95, 4.52
Rich				
**Constant**	−31.44	102.63	0.759	−233.00, 170.11
**Sigma**	248.45	9.46		229.87, 267.03

CI, confidence interval; HBV, hepatitis B virus; SE, standard error.

Moreover, the marginal effect of the variable, sex, revealed that male participants were willing to pay approximately ETB 34.37 (95% CI = 6.34, 62.40) higher than females. As the age of the household head increased by 1 year, the amount of money willing to pay for the HBV vaccine was reduced by ETB 3.36 (95%CI = −4.51, −2.20). Moreover, single and divorced/widowed were willing to pay ETB 40.13 (95%CI = −69.55, −10.71) and ETB 38.19 (95%CI = −68.29, −8.10) lower than married individuals, correspondingly. Whereas, as the score of attitude and knowledge toward HBV infection and vaccine increased by one the estimated mean of the marginal effects was 6.76 (95%CI = 1.24, 12.27) and 27.11 (95%CI = 18.52, 35.70), respectively (Table 4).

**Table 4:** Marginal effects of factors influencing WTP for HBV vaccine.

**Variables**	**dy/dx**	**SE**	***P*-value**	**95% CI**
**Sex**				
Male	34.37	14.30	0.016	6.34, 62.40
Female				
**Age**	−3.36	0.59	0.000	−4.51, −2.20
**Family history of HBV infection**				
Yes	26.35	18.72	0.159	−10.34, 63.04
No				
**Information about HBV**				
Yes	−21.36	18.69	0.253	−57.99, 15.27
No				
**Tattoo on the body**				
Yes	−23.73	15.21	0.119	−53.55, 6.08
No				
**Attitude**	6.76	2.81	0.016	1.24, 12.27
**Knowledge**	27.11	4.38	0.000	18.52, 35.70
**Marital status**				
Single	−40.13	15.01	0.008	−69.55, −10.71
Divorced/widowed	−38.19	15.36	0.013	−68.29, −8.10
Married				
**Education status**				
Primary	−17.11	27.12	0.528	−70.26, 36.04
Secondary	−29.55	26.99	0.274	−82.46, 23.36
Diploma	−42.45	28.37	0.135	−98.04, 13.15
Degree and above	8.11	31.07	0.794	−52.79, 69.01
Unable to read and write				
**Wealth status**				
Poor	19.32	17.70	0.275	−15.36, 54.01
Medium	−25.23	14.22	0.076	−53.11, 2.64
Rich				

CI, confidence interval; HBV, hepatitis B virus; SE, standard error.

## Discussion

The current study aimed to assess the willingness to pay for the HBV vaccine and its associated factors among households in Bahir Dar City. The findings showed that the majority of the participants were willing to pay for the HBV vaccine, and sex, age, marital status, knowledge, and attitude toward the HBV infection were significantly associated with WTP for the vaccine.

In this study, 62.0% of participants were willing to pay for the HBV vaccine. The percentage of participants who were willing to pay is comparable with the study conducted in Gondar, Ethiopia (14). This might be related to the information that the households have toward the infection and vaccine as in the current study 76.81% had information about HBV infection. According to another study (33), a comparable number (60.6%) of households were willing to pay for childhood malaria vaccines among caregivers of under-five children in Northwest Ethiopia (33). On the other hand, the proportion of households that are willing to pay for the HBV vaccine in this study is lower than the 83.4% proportion of households that are willing to pay for cervical cancer screening (34). The number of participants who were willing to pay for the HBV vaccine is higher than in the study conducted in Malaysia (11) where only 37.5% of the households were willing to pay for the HBV vaccine. However, it is lower than the study conducted in Vietnam (13), where 80.8% of the reproductive-age women were willing to pay for the HBV vaccine. The discrepancy might be attributable to the socioeconomic, study participants and sample size differences among the countries.

The study also revealed that the mean amount of money HHs willing to pay was ETB 174.24 (US$5.25) ± 145.44 ($4.38). This average amount of money that individuals were willing to pay was lower than the market price for adult HBV vaccination which ranges from US$65-US$134 (35) and the study conducted in Gondar, Ethiopia (14), where the average cost of HBV vaccine that the participants willing to pay were ETB 325.83 (US$14.39). This might be because participants in the previous study were healthcare professionals who have financial security as government employees than the population of this study that most of them are non-government employees. The amount of money that the households were willing to pay in this study was lower than the US$596 that the population group with the worst health status in Iran was willing to pay for health care (36).

The current study showed that marital status was statically associated with WTP: single and divorced/widowed were willing to pay ETB 40.13 and ETB 38.19 less than married individuals. The possible explanation for this might be that a widowed HH head might have a high economic burden than a married one as there is no someone to accompany in household expenditure. A study conducted elsewhere (37) also indicated that widowhood is associated with a higher risk of economic deprivation. Besides, consistent with other studies (38–40), male HH heads were willing to pay ETB 34.37 higher for the HBV vaccine than female HH heads. This could be due to males often predominantly dealing with big financial matters as compared to a female who usually deals with recurrent household expenditures in Ethiopian settings. On the other hand, there was also a statistically significant negative relationship between respondents' age and their WTP. As indicated by other scholars (38, 40, 41), as the age of the household head increased by 1 year, the amount of money that the household head was willing to pay for the HBV vaccine was reduced by ETB 3.36. This might be due to the low information toward the presence and benefits of the vaccine among adults as compared to the young population which may affect their willingness. This was also supported by the finding that the respondents' attitudes and knowledge about HBV infection and vaccines were positively associated with WTP. In this study as the knowledge and attitude scores of the respondents increased by one their willingness to pay for the HBV vaccine will be increased by 6.76 and 27.11, respectively. The findings are consistent with other studies (29, 42–44).

Despite its strengths such as community-based study and employing the contingent valuation method, some limitations need to be considered in interpreting and concluding the results. First, in applying contingent valuation techniques, a possible source of bias might arise from the fact that respondents are not purchasing the vaccine in the practical context but rather hypothetically. Second, the cross-sectional analysis does not allow for the establishment of a causal relationship between the explained and explanatory variables. Finally, even if this finding is promising, it should be explored with another study design such as a discrete choice experiment and needs to cover large settings to conclude for the national level.

## Conclusion

Overall, most of the Bahir Dar city households were willing to pay for the HBV vaccine. This showed households demanded the vaccine and the government need to put the HBV vaccine into the national public health strategy. Moreover, attitudes and knowledge were positively associated with willingness to pay. Therefore, strategies should be considered to enhance the household's attitude toward the HPV vaccine, and knowledge level and information about the vaccine should be provided through various modalities such as mass media and campaigns. However, age and female household heads were willing to pay less price for the HBV vaccine compared to their counterparts. As a result, policymakers should consider this difference in the ability to pay for the vaccine while developing a program to cover these population groups.

## Data availability statement

The raw data supporting the conclusions of this article will be made available by the authors, without undue reservation.

## Ethics statement

The studies involving human participants were reviewed and approved by an ethical clearance letter (Ref No/IPH/837/6/2020) was obtained from the Ethical Review Committee of the Institute of Public Health, University of Gondar. The patients/participants provided their written informed consent to participate in this study.

## Author contributions

AA conceived the study, developed data collection tools, performed the analysis and interpretation of data, and obtained research funding. AM, CT, TA, and AYA participated in the development of the study proposal, analysis, and interpretation. AA, CT, and TA drafted the original manuscript. AYA revised the manuscript. All authors have seen and approved the final version of the manuscript for submission.
